# Clinical characteristics and outcomes of critically ill patients with acute COVID-19 with Epstein-Barr virus reactivation

**DOI:** 10.1186/s12879-021-06638-y

**Published:** 2021-09-15

**Authors:** Yun Xie, Song Cao, Hui Dong, Hui Lv, Xiaolei Teng, Jiaxiang Zhang, Tao Wang, Xiaoyan Zhang, Yun Qin, Yujing Chai, Luyu Yang, Jun Liu, Ruilan Wang

**Affiliations:** 1 Critical Care Unit, Shanghai General Hospital, Shanghai Jiao Tong University School of Medicine, 650 New Songjiang Road, Songjiang, Shanghai, 201600 People’s Republic of China; 2 Critical Care Unit, Wuhan Third Hospital, Wuhan University, Wuhan, China; 3 Chongming Hospital of the Branch Xinhua Hospital, Shanghai Jiao Tong University School of Medicine, Shanghai, China; 4 Shanghai Yangpu District Mental Health Center, Shanghai, China; 5 Nephrology, Shanghai General Hospital, Shanghai Jiao Tong University School of Medicine, Shanghai, China

**Keywords:** Epstein-Barr virus, SARS-CoV-2, Outcome

## Abstract

**Background:**

Our goal is to further elucidate the clinical condition and prognosis of patients with severe acute COVID-19 with EBV reactivation.

**Method:**

This is a retrospective single-center study of COVID-19 patients admitted to the intensive care unit of Wuhan No. 3 Hospital (January 31 to March 27, 2020). According to whether Epstein-Barr virus reactivation was detected, the patients were divided into an EBV group and a Non-EBV group. Baseline data were collected including epidemiological, larithmics, clinical and imaging characteristics, and laboratory examination data.

**Results:**

Of the 128 patients with COVID-19, 17 (13.3%) were infected with Epstein-Barr virus reactivation. In the symptoms,the rate of tachypnoea in the EBV group was apparently higher than that in the Non-EBV group. In lab tests, the lymphocyte and albumin of EBV group decreased more significantly than Non-EBV group, and the D-dimer and serum calcium of EBV group was higher than Non-EBV group. Regarding the infection index, CRP of EBV group was apparently above the Non-EBV group, and no significant difference was found in procalcitonin of the two groups. The incidence of respiratory failure, ARDS, and hypoproteinaemia of EBV group had more incidence than Non-EBV group. The 28-day and 14-day mortality rates of EBV group was significantly higher than that of Non-EBV group.

**Conclusions:**

In the COVID-19 patients, patients with EBV reactivation had higher 28-day and 14-day mortality rates and received more immuno-supportive treatment than patients of Non-EBV group.

## Background

Severe acute respiratory syndrome coronavirus 2 (SARS-CoV-2) pneumonia is a highly contagious disease that still spreads widely around the world, COVID-19 is still in pandemic form around the world. Although most patients have a favorable evolution, some patients progress to acute respiratory failure and acute respiratory distress syndrome (ARDS), even other dangerous complications developed very quickly, culminating in exacerbation and death [1, 2]. The mortality rate in severe cases of SARS-COV-2 is acknowledged to be high, which reported 61.5% [3].

Epstein-Barr virus (EBV), also known as human herpesvirus IV, is herpesvirus only infects humans and a few primates. After entering the human body, EBV mainly invades B lymphocytes, T lymphocytes, epithelial cells and muscle cells [4]. In EBV infection, the virus can become latent (inactive). Critically ill patients are more likely to have activation.

Currently, there is very little information about EBV infection in patients with COVID-19. Specific information describing the characteristics of EBV in critically ill patients remains unknown. In this study, we investigated cases of COVID-19 coinfected with EBV.

## Methods

### Study design and participants

Our study was conducted as a retrospective, single-center, observational research at the Third Hospital in Wuhan, China (COVID-19 sentinel hospital) (January 31 to March 27, 2020). Ethics Committee of Wuhan Third Hospital, Wuhan, China, approved the study, Due to the rapid spread of the epidemic and retrospective studies, written informed consent was exempted. The institutional review board approved the decision to waive consent. Access to the raw data/samples did not require administrative privileges. Data used in this study were anonymized prior to use. The diagnostic criteria for COVID-19 was the World Health Organization (WHO) interim guidelines [5]. EBV deoxyribonucleic acid (DNA) ≥ 500 copies/mL, positive EBV capsid antibody immunoglobulin M (IgM) or EBV early antibody IgM/immunoglobulin G (IgG) [6] was diagnosed EBV infection. Critically ill patients were defined as those in the intensive care or critical care unit.

### Inclusion criteria were as follows

Patients with definite diagnosis of COVID-19, COVID-19 was diagnosed by real-time reverse transcription polymerase chain reaction (RT-PCR) testing.

Exclusion criteria were as follows: Patients with incomplete clinical data collection.

The criteria for severe disease were as follows [7, 8]: One of the following was met:

1. Patient was tachypneic with RR ≥ 30 breaths/min;
2. Patient's resting oxygen saturation (SpO_2_) ≤ 93%;
3. Arterial partial pressure of oxygen/inhaled oxygen ≤ 300 mmhg;
  Altitude > 1000 m should be corrected:PaO2/FiO2 × [air pressure (mmHg)/760]; or
4. Patient's lung images show significant progression of lung lesions, ≥ 50% within 24–48 h.

Criteria for critical illness were as follows [7, 8]: Meet one of the following:

1. The patient is in respiratory failure and requires mechanical ventilation;
2. The patient is in shock;
3. The patient has other organ failure and requires ICU care.

### Data collected

COVID-19 exposure epidemiology, demographics, vital signs on admission, clinical, laboratory, administrative and clinical outcomes data were obtained from the patients' hospital charts. In turn, these data were compared between EBV reactivated patients and non-EBV reactivated patients.

Upper respiratory tract swabs (mouth swab, throat swab, nose swab) of patients at admission were preserved in virus preservation solution. SARS-CoV-2 nucleic acid was detected through RT-PCR. In addition, chest x-ray or chest CT was performed in all patients with suspected COVID-19.

Mortality at 28 days after admission was the primary outcome of the study. 14-day mortality was the secondary outcome of the study.

Treatment regimen [7, 8]: all patients received oxygen therapy (nasal cannula, mask, High-flow oxygen inhalers, non-invasive/invasive ventilator, or even prone ventilation) and empirical antiviral therapy according to oxygenation, with initial administration of moxifloxacin antibiotic therapy and subsequent antibiotic therapy adjusted on the basis of the patient's changing signs and symptoms and lab bacterial fungal culture. In addition, depending on the patient's elevated d -dimer or detection of venous thrombosis, low molecular weight heparin calcium injection was administered, if the patient's lymphocyte dropped to < 0.5 × 109/L, intravenous 20 g/d immunoglobulin (IVIG) was given. If the absolute lymphocyte count remained low after 5 days, thymidine was administered.Critically ill cases were given 1–2 mg/kg intravenous glucocorticoids for 5–7 days. If patients had hypoproteinemia, they received intravenous albumin infusion therapy. WHO guidelines were followed for all other treatments [5].

### Statistical analysis

If the data is normally distributed, we used continuous measurements as mean (Standard Deviation, SD), independent sample T test was used to compare the differences between the two groups of sample data. If the data is not normally distributed, median (interquartile range) and non-parametric tests were used to compare differences. Chi-square test or Fisher's test statistics were used for counting data. We used Kaplan–Meier plots to analyze survival data. EBV and non-EBV groups were compared using a stratified logrank test and a stratified multivariate Cox proportional risk model with lymphocyte count as a stratification factor. Statistical analysis was performed using SPSS 13.0 (SPSS Inc.).

## Results

**Fig. 1:**
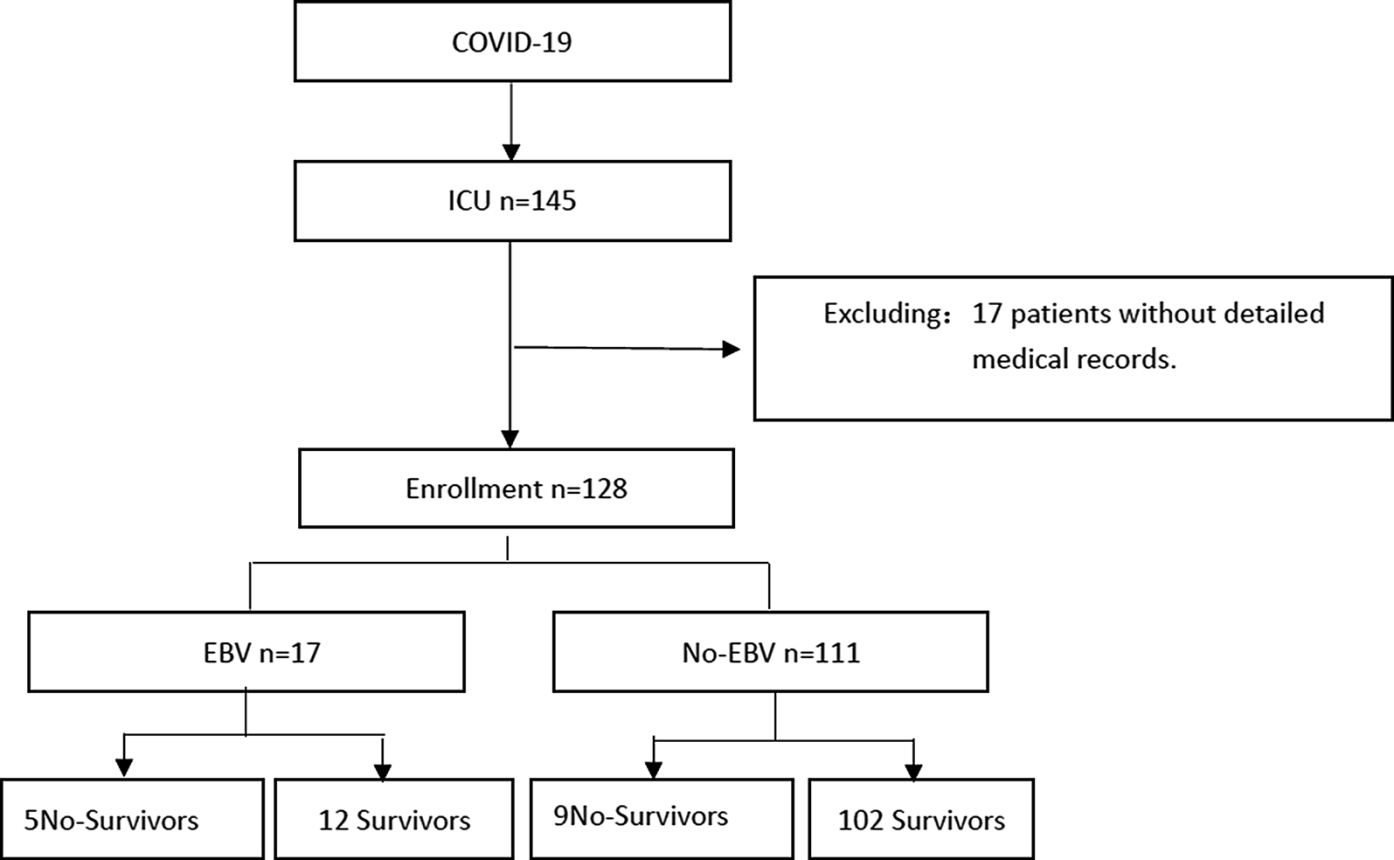
Research flow chart

By March 27, 2020, 1516 patients had been admitted to Wuhan Third Hospital with confirmed COVID-19, and 145 (9.6%) patients were got to the ICU.17 patients without detailed medical records were excluded. Our study included 128 critically ill COVID-19 patients. Of these, 66 (51.6%) were male, average age of 62 years old (IQR52-68). Fourteen patients (10.9%) deteriorated and the cause of death was multiple organ failure within 28 days of admission (Fig. 1). At admission, 53 (41.4%) cases had tachypnoea. 8 patients (6.3%) with low platelet counts. Abnormal liver function (Alanine aminotransferase and alanine aminotransferase were higher than normal levels) was found in 11 (8.6%) patients. There were 11 cases with an abnormal myocardial enzyme spectrum and 12 cases (9.4%) with acute kidney injury. A total of 17 (13.3%) patients were diagnosed with EBV infection.

According to whether they were infected with EBV reactivation, two groups were divided, EBV group (n = 17) and Non-EBV group (n = 111). In symptoms, the frequency of tachypnoea in the EBV group was significantly greater than that in the Non-EBV group. In lab tests, lymphocyte and albumin were lower in the EBV group, D-dimer and serum calcium were lower in the Non-EBV group. EBV group had significantly higher C-reactive protein (CRP) and no statistical difference was found in procalcitonin between the two groups. The incidence of respiratory failure was significantly more in the EBV group than in the Non-EBV group (76.5% vs 33.3%, p = 0.001), ARDS (88.2% vs 34.2%, p < 0.001). The incidence of hypoproteinaemia was higher in EBV group (88.2% vs 39.6%, p < 0.01).No significant difference in acute myocardial injury, acute liver injury, acute kidney injury, sepsis, heart failure, septic shock, coagulopathy, secondary infection, acidosis between the two group.EBV group had significantly higher 28-day and 14-day mortality than Non-EBV group (Table 1).

**Table 1:** Demographic characteristics, baseline characteristics, and clinical outcomes of patients with SARS-CoV 2 infection

	All patients n = 128	EBV n = 17	Non-EBV n = 111	P value
Demographic characteristics, baseline characteristics				
Age, years	62 (52–68)	62 (51.5–72.5)	61 (52–66)	0.768
Sex				
Men	66 (51.6)	10 (58.8)	56 (50.4)	0.520
Women	62 (48.4)	7 (41.2)	55 (49.6)	0.520
Symptoms				
Respiratory rate	25 (20–41)	25 (20–47.5)	25 (22.25–41.25)	0.849
> 24 breaths per min	53 (41.4)	15 (88.2)	38 (34.2)	< 0.001*
Systolic pressure, mmHg	133 (120–147)	127(116–152.25)	138 (122–148)	0.820
SpO_2_,%	93 (82–97)	95 (73.5–98.75)	92 (82–95)	0.752
Heart rate	94 (86–99)	86 (84.5–106.5)	95 (93–100)	0.611
Laboratory test				
White blood cell count, × 10^9^/L	8.4 (4.6–10.8)	10.35 (7.925–13.25)	7.1 (4–9.1)	0.081
Neutrophil %	89.2 (82.2–92)	91.2 (88.2–92.3)	88.9 (72.7–91.6)	0.288
Lymphocyte count, × 10^9^/L	0.58 (0.4–0.67)	0.54 (0.335–0.6525)	0.59 (0.4–0.75)	0.0002**
Lymphocyte%	6.14 (4.73–12.79)	5.013 (3.92–6.77)	6.42 (5.61–15.59)	0.278
Platelet < 100 × 10^9^/L, %	8 (6.25)	2 (11.7)	6 (5.41)	0.313
D-dimer, mg/L	6.07 (2.26–6.9)	6.67 (1.85-32.9)	4.26 (2.26–6.39)	< 0.0001**
Fibrinogen, g/L	5.81 (4.92–6.32)	6.15 (5.6-6.52)	4.93 (4.625–6.23)	0.177
Albumin, g/L	27.3 (25.2–29.7)	25.6 (22.65–28.42)	28.2 (26.9–30.2)	0.03*
Potassium, mmol/L	3.6 (3.22–3.9)	3.6 (3.2–4.0)	3.515 (3.255–3.877)	0.910
Calcium, mmol/L	1.81 (1.14–1.92)	1.91 (1.855–2)	1.18 (1.13–1.9)	< 0.001*
Sodium, mmol/L	142 (141–145)	141 (140–141)	142 (141–145)	0.743
Procalcitonin, µg/L	0.34 (0.135–0.88)	0.475 (0.31–1.72)	0.12 (0.07-0.12)	0.257
C-reactive protein, mg/	121.8 (42.31–259.6)	262.4 (122.3–271)	53.9 (17.79–137)	0.004*
pH	7.42 (7.39–7.49)	7.46 (7.39–7.46)	7.41 (7.385–7.47)	0.347
PaO_2_, mmHg	54(48.5-150.5)	49.0 (43–106)	57 (49.5–170)	0.432
PaCO_2,_ mmHg	38.2 (34.4–42.15)	34.2(28.6–47.82)	38.8 (37.75–42.15)	0.483
Lac, mmol/L	1.05 (0.625–1.65)	1 (0.6–1)	1.1 (0.6–1.85)	0.821
HCO_3_^–^	24.75 (23.65–29.15)	26.2 (22.8–26.2)	24.1 (23.7–29)	0.726
Clinical outcomes				
Acute myocardial injury%	11 (8.59)	2 (11.76)	9 (8.11)	0.640
Acute liver injury%	11 (8.59)	2 (11.76)	9 (8.11)	0.640
Acute kidney injury%	12 (9.37)	2 (11.76)	10 (9.01)	0.661
Sepsis%	10 (7.81)	2 (11.76)	8 (7.21)	0.621
Respiratory failure%	50 (39.1)	13 (76.5)	37 (33.3)	0.001*
ARDS%	53 (41.4)	15 (88.2)	38 (34.2)	< 0.001*
Heart failure%	4 (3.12)	1(5.88)	3 (2.71)	0.439
Septic shock%	4 (3.12)	0	4 (3.60)	1
Coagulopathy%	1 (0.781)	1 (5.88)	0	0.133
Secondary infection%	2 (1.56)	1 (5.88)	1 (0.90)	0.249
Hypoproteinaemia%	59 (46.1)	15 (88.2)	44 (39.6)	< 0.001*
Acidosis%	3 (2.34)	1 (5.88)	2 (1.80)	0.350
28-day mortality%	14 (10.9)	5 (29.4)	9 (8.11)	0.0046*
14-day mortality%	10 (7.81)	5 (29.4)	5 (4.5)	0.0046*

Data are presented as the median (IQR), n (%),*p < 0.05. SARS-CoV-2, Severe acute respiratory syndrome coronavirus 2; EBV, Epstein-Barr virus; SpO2, saturation of pulse oximetry; pH, potential of hydrogen; PaO2, arterial partial oxygen pressure; PaCO2, partial pressure of carbon dioxide in arterial blood; Lac, lactic acid; ARDS, acute respiratory distress syndrome

**Fig. 2:**
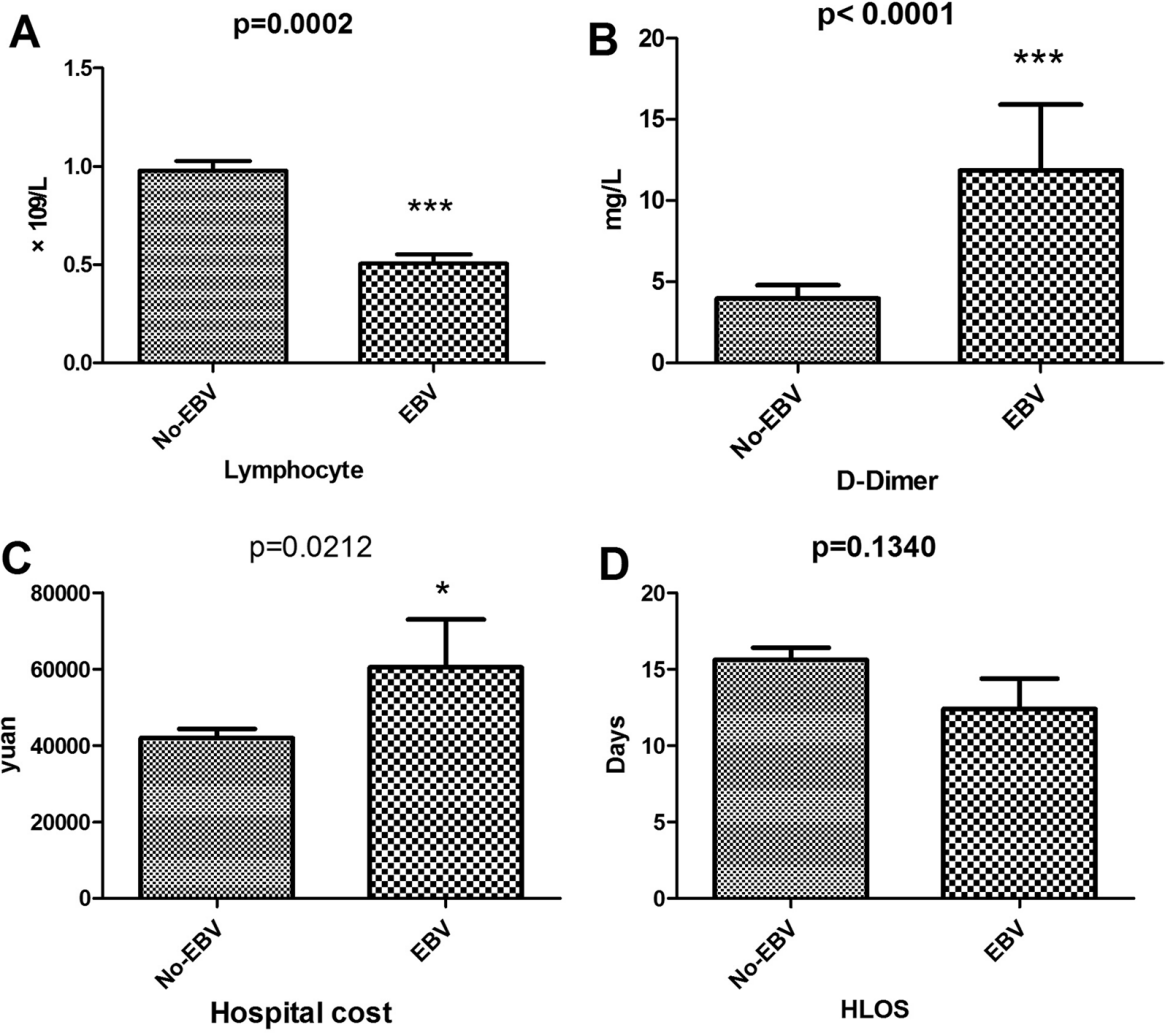
**A**. Lymphocyte counts of the two groups (**B**). D-dimer levels of the two groups (**C**). Hospital costs of the two groups (**D**). Hospital lengths of stay of the two groups

**Fig. 3:**
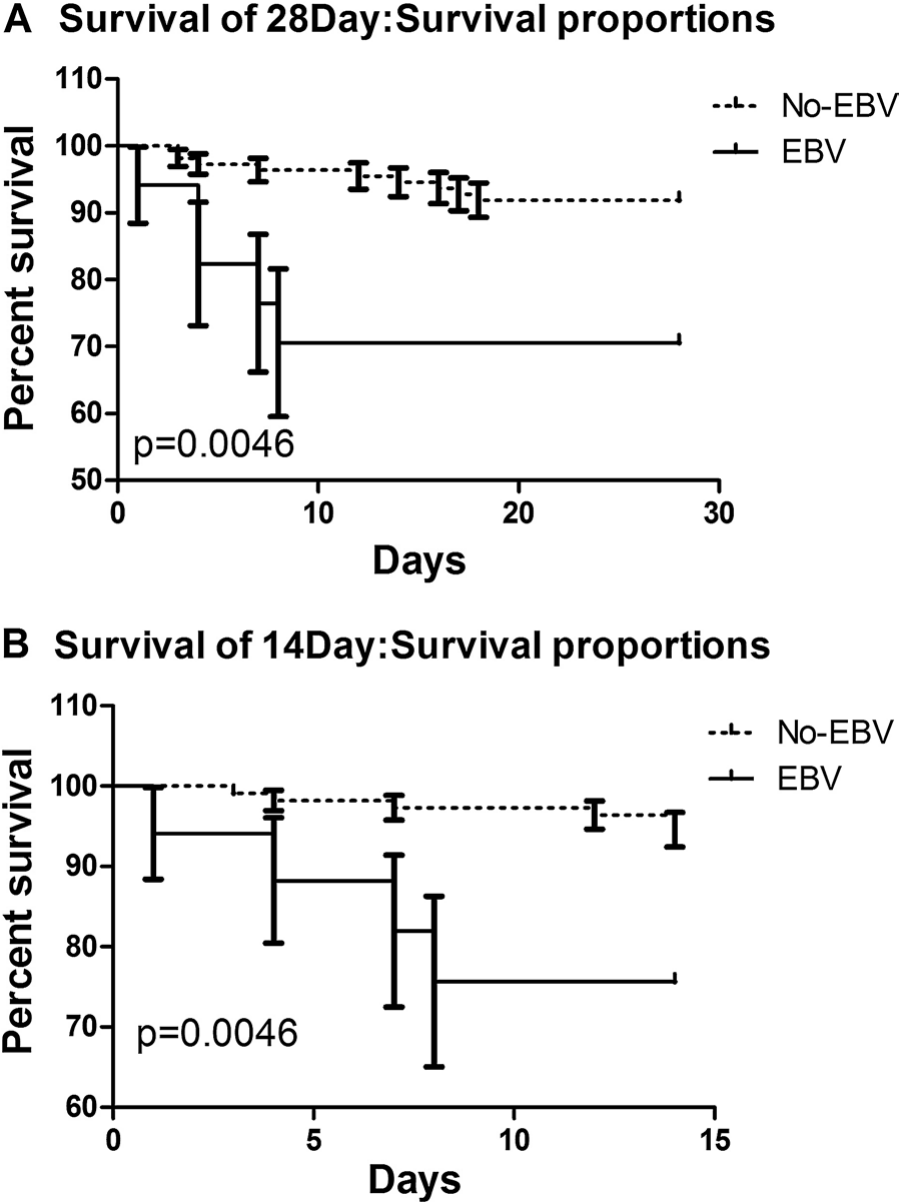
**A** 28-Day survival proportions **B** 14-Day survival proportions

**Fig. 4:**
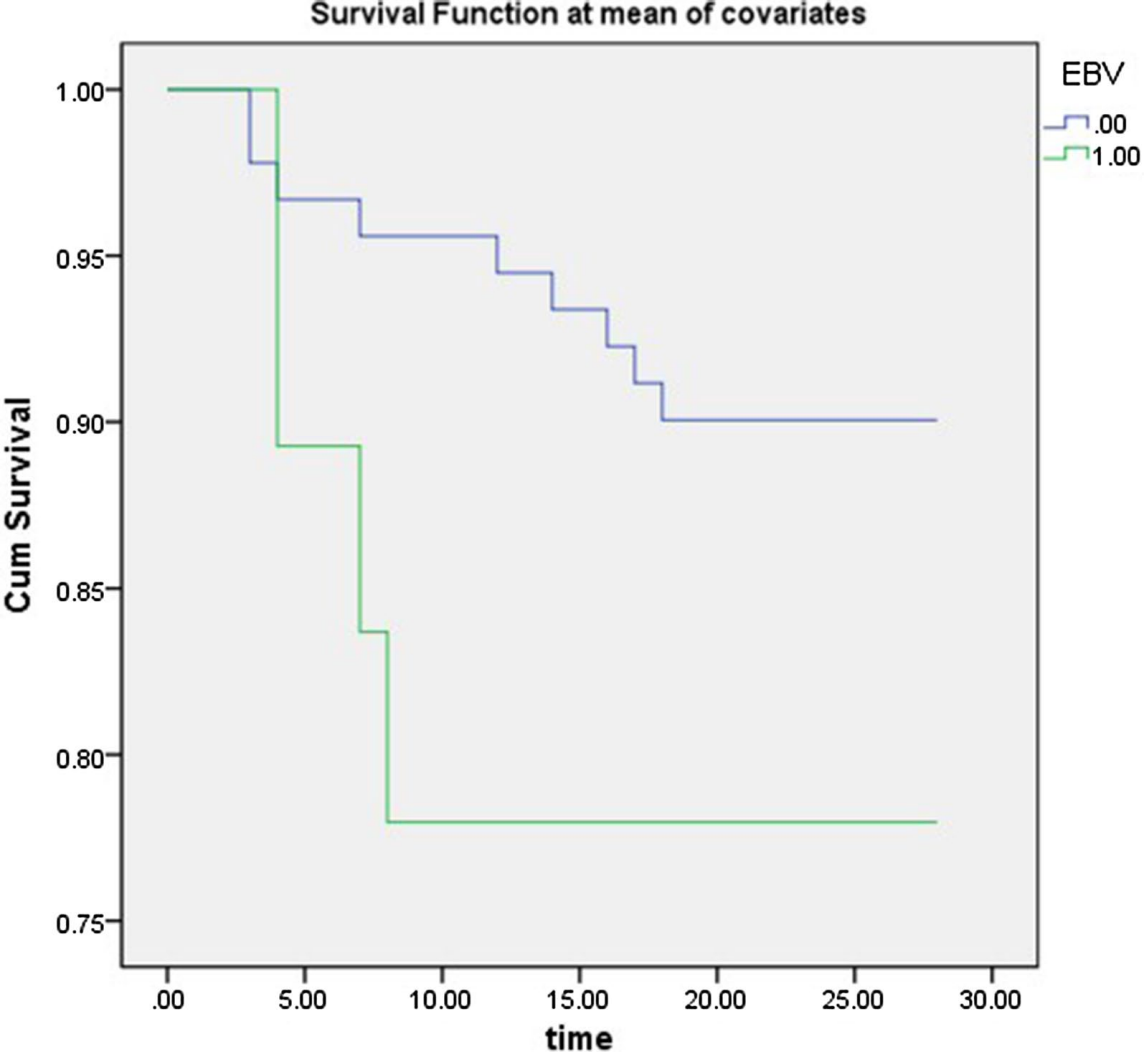
28-Day survival proportions of Cox model

There were no significant differences in the hospital stay or hospital cost between the two groups (Fig. 2). Figure 3A and B also showed that Non-EBV group had significantly higher 28-day and 14-day survival proportions than EBV group (p = 0.0046 by log-rank test). When the Lymphocyte count was taken into account, 28-day survival continued to be significantly shorter in the EBV group than in the non-EBV group (P < 0.001), according to the stratitized Logunk test. A multivariate analysis showed that the prognosis was significantly better in the non-EBV group (hazard ratio [HR], 0.560; 95% CI 0.116–2.689; P < 0.001) (Fig. 4).

## Discussion

As an observational and monocentric study of COVID-19 with EBV reactivation, it includes data from 128 patients in Wuhan Third Hospital. This study presents the COVID-19 with EBV reactivation had higher 28-day and 14-day mortality rates and received more immuno-supportive treatment.

The 28-day mortality rate (10.9%) and 14-day mortality rate (7.8%) of critically ill COVID-19 patients were similar to those of a previous study [3, 9], but patients with EBV reactivation had increased mortality (29.4%).

Of the 128 cases of COVID-19, we observed more severe symptoms and higher rates of tachypnoea in patients with EBV reactivation. Patients with EBV reactivation had more severe illness, with significantly lower lymphocytes and higher D-dimers. Sustained lymphocytic decline and D-dimer increase are indicative of worsening of the disease. In the acute stage of SARS-COV infection, peripheral blood lymphocytes, mainly T lymphocytes, were rapidly reduced [10], and CD4+ and CD8+ T lymphocytes were also reduced. Lymphocyte loss may predates abnormal chest X-ray changes [11, 12]. The ratio of lymph cells to white blood cells can also validate that the immune function of COVID-19 patients with EBV reactivation is poor, so we suggest treatment to improve immune function to promote recovery. We used IVIG in combination with LMWH to treat COVID-19, particularly in cases with EBV reactivation, in accordance with Lin et al. [13]. In our study, we found that EBV group had higher 28-day and 14-day mortality rates than Non-EBV group. Hyperinflammatory response was leaded by SARS-CoV-2 and then EBV infection or reactivion, which leads to a worse prognosis. As the number of cases of this virus increases, so do the number of complications exposed. Chronic B-lymphocyte depletion ultimately puts patients at higher risk for infections other than SARS-CoV-2 [14]. EBV reactivation was closely related to the prognosis of patients with COVID-19, which may exacerbate COVID-19 pneumonia. We believed that this was mainly related to the outbreak of inflammatory cytokines after EBV reactivation, which still needs to be confirmed by further mechanism studies. EBV and Non-EBV groups had no significantly difference in the HLOS and hospital costs, which might be because patients who died had shorter hospital stays and lower costs of hospitalization.

Our study also found that group with EBV reactivation had higher serum calcium than Non-EBV group, possibly because latent membrane protein 1 increased calcium inflow through the store-operated channel in B-lymphoid cells [4]. The patients in our study with EBV infection had higher CRP levels. It has been reported that CRP can reflect the severity of EBV infection, chronic fatigue and chronic fatigue syndrome are triggers of acute EBV infection. A previous research found that CRP was the baseline predictor of EBV infection after six months chronic fatigue [15]. The CRP/albumin ratio is a valuable coadjutant for EBV DNA levels for identifying survival differences [16]. Moreover, we found that patients with EBV reactivation had lower albumin, which also suggests that the disease in these patients is more severe.

There are several limitations in this study. First, our research is monocentric and retrospective. Such studies are rare in the literature, and in the future we will include multiple centers to collect more cases to more fully understand COVID-19. At the same time, more detailed data, particularly the inflammatory cytokines, is not included in the analysis. However, the information in this research can be used for the early understand of EBV reactivation in COVID-19 pneumonia of China.

## Conclusion

In conclusion, it is not clear if COVID-19 patients are susceptible to new infections of EBV or to reactivation of latent EBV, but patients with EBV co-infection have lower lymphocytes, higher D-dimer levels, more severe symptoms, and higher 28-day and 14-day mortality and require more immuno-supportive treatments, such as IVIG, than do patients without EBV.

## Data Availability

After publication, the data will be made available to others on reasonable request to the corresponding author.

## Abbreviations

IVIG: Intravenous immunoglobulin
COVID-19: Coronavirus disease-19
ICU: Intensive care unit
CT: Computed tomography
EBV: Epstein-Barr virus
SARS: Severe acute respiratory syndrome
RT-PCR: Reverse transcription-polymerase chain reaction
HLOS: Hospital length of stay
ICULOS: Intensive care unit length of stay
LMWH: Low molecular weight heparin

## References

[1] Yu P, Zhu J, Zhang Z (2020). A familial cluster of infection associated with the 2019 novel coronavirus indicating potential person-to-person transmission during the incubation period. J Infect Dis..

[2] Chen N, Zhou M, Dong X (2020). Epidemiological and clinical characteristics of 99 cases of 2019 novel coronavirus pneumonia in Wuhan, China: a descriptive study. Lancet..

[3] Yang X, Yu Y, Xu J (2020). Clinical course and outcomes of critically ill patients with SARS-CoV-2 pneumonia in Wuhan, China: a single-centered, retrospective, observational study. Lancet Respir Med.

[4] Dellis O, Arbabian A, Papp B (2011). Epstein-Barr virus latent membrane protein 1 increases calcium influx through store-operated channels in B lymphoid cells. J Biol Chem.

[5] WHO. Clinical management of severe acute respiratory infection when Novel coronavirus (nCoV) infection is suspected: interim guidance. 2020. https://www.who.int/internal-publications-detail/clinical-management-of-severe-acute-respiratory-infection-when-novel-coronavirus-(ncov)-infection-is-suspected.

[6] Okano M, Kawa K, Kimura H (2005). Proposed guidelines for diagnosing chronic active Epstein-Barr virus infection. Am J Hematol.

[7] Xie Y, Cao S, Dong H (2020). Effect of regular intravenous immunoglobulin therapy on prognosis of severe pneumonia in patients with COVID-19. J Infect..

[8] Xie Y, Dong H, Liao Y (2021). A prediction model of mortality in COVID-19 pneumonia based on CT score and lymphocyte count. Austin J Pathol Lab Med.

[9] Deng YY, Lu PX, Yang GL (2010). Correlative study of semi-quantitative score of chest CT findings and viral load in novel influenza A (H1N1)virus infection. Radiol Practi ce.

[10] Taisheng L, Zhifeng Q, Linqi Z (2004). Significant changes of peripheral T lymphocyte subsets in patients with severe acute respiratory syndrome. J Infect Dis.

[11] Liu ZY, Li TS, Wang Z (2003). Clinical features and therapy of 106 cases of severe acute respiratory syndrome. Chin J Intern Med.

[12] Li TS, Qiu ZF, Han Y (2003). The alterations of T cell subsets of severe acute respiratory syndrome during acute phase. Chin J Lab Med.

[13] Lin L, Lu L, Cao W (2020). Hypothesis for potential pathogenesis of SARS-CoV-2 infection–a review of immune changes in patients with viral pneumonia. Emerg Microbes Infect..

[14] Amir R, Kichloo A, Singh J (2020). Epstein-Barr virus versus novel coronavirus-induced hemophagocytic lymphohistocytosis: the uncharted waters. J Investig Med High Impact Case Rep.

[15] Pedersen M, Asprusten TT, Godang K (2019). Predictors of chronic fatigue in adolescents six months after acute Epstein-Barr virus infection: a prospective cohort study. Brain Behav Immun.

[16] Shasha H, Yan W, Haiyang C (2016). C-Reactive Protein/Albumin Ratio (CAR) as a prognostic factor in patients with non-metastatic nasopharyngeal carcinoma. J Cancer.

